# ICA-based denoising for ASL perfusion imaging

**DOI:** 10.1016/j.neuroimage.2019.07.002

**Published:** 2019-10-15

**Authors:** D. Carone, G.W.J. Harston, J. Garrard, F. De Angeli, L. Griffanti, T.W. Okell, M.A. Chappell, J. Kennedy

**Affiliations:** a Acute Vascular Imaging Centre, Radcliffe Department of Medicine, University of Oxford, Oxford, United Kingdom; b Laboratory of Experimental Stroke Research, Department of Surgery and Translational Medicine, University of Milano Bicocca, Milan Center of Neuroscience, Monza, Italy; c Wellcome Centre for Integrative Neuroimaging, FMRIB, Nuffield Department of Clinical Neurosciences, University of Oxford, United Kingdom; d Institute of Biomedical Engineering, Department of Engineering Science, University of Oxford, United Kingdom

**Keywords:** Arterial spin labeling, ASL, Independent component analysis, ICA, SNR, Denoising

## Abstract

Arterial Spin Labelling (ASL) imaging derives a perfusion image by tracing the accumulation of magnetically labeled blood water in the brain. As the image generated has an intrinsically low signal to noise ratio (SNR), multiple measurements are routinely acquired and averaged, at a penalty of increased scan duration and opportunity for motion artefact. However, this strategy alone might be ineffective in clinical settings where the time available for acquisition is limited and patient motion are increased. This study investigates the use of an Independent Component Analysis (ICA) approach for denoising ASL data, and its potential for automation.

72 ASL datasets (pseudo-continuous ASL; 5 different post-labeling delays: 400, 800, 1200, 1600, 2000 m s; total volumes = 60) were collected from thirty consecutive acute stroke patients. The effects of ICA-based denoising (manual and automated) where compared to two different denoising approaches, aCompCor, a Principal Component-based method, and Enhancement of Automated Blood Flow Estimates (ENABLE), an algorithm based on the removal of corrupted volumes. Multiple metrics were used to assess the changes in the quality of the data following denoising, including changes in cerebral blood flow (CBF) and arterial transit time (ATT), SNR, and repeatability. Additionally, the relationship between SNR and number of repetitions acquired was estimated before and after denoising the data.

The use of an ICA-based denoising approach resulted in significantly higher mean CBF and ATT values (p < 0.001), lower CBF and ATT variance (p < 0.001), increased SNR (p < 0.001), and improved repeatability (p < 0.05) when compared to the raw data. The performance of manual and automated ICA-based denoising was comparable. These results went beyond the effects of aCompCor or ENABLE. Following ICA-based denoising, the SNR was higher using only 50% of the ASL-dataset collected than when using the whole raw data.

The results show that ICA can be used to separate signal from noise in ASL data, improving the quality of the data collected. In fact, this study suggests that the acquisition time could be reduced by 50% without penalty to data quality, something that merits further study. Independent component classification and regression can be carried out either manually, following simple criteria, or automatically.

## Highlights

• ICA can be used to separate signal from noise in ASL data, improving data quality.
• Automated denoising reproduces the improvement seen by a manual approach.
• ICA based denoising is superior to PCA or volume censoring approaches.
• ASL acquisition time could be reduced by 50% without penalty to data quality.

## 1 Introduction

The measurement of cerebral perfusion is an indispensable tool in clinical practice across a broad range of acute and chronic pathologies, such as stroke and dementia (Grade et al., 2015; Albers et al., 2018; Wolters et al., 2017). A number of methodologies can be used, each with its own advantages and disadvantages; arterial spin labeling (ASL) (Detre et al., 1992) MRI’s key advantage is that it does not require the administration of an exogenous contrast agent. Instead, ASL generates an image by tracing the accumulation of magnetically labeled blood water in the brain, deriving a perfusion image by subtracting a magnetically labeled image from a control unlabeled image. The use of multiple post-labeling delays (PLD) in the acquisition allows the estimation of arterial transit time (ATT) values, which may not only improve the accuracy of the quantification of cerebral blood flow (CBF) (Wang et al., 2013; Okell et al., 2013), but may also provide relevant risk stratification information, for instance, in patients with carotid steno-occlusive arterial disease (Alsop et al., 2015).

The main drawback of ASL is that the generated image has an intrinsically low signal to noise ratio (SNR). Multiple measurements are routinely acquired and averaged to compensate for this (Alsop et al., 2015), though at a penalty of increased scan duration and, hence, opportunity for motion artefacts, whose adverse effect are further enhanced by the image subtraction process (Grade et al., 2015). Thus, in acute clinical settings, patient factors, and, in particular, increased patient movement (Carone et al., 2017) may limit this strategy as a means of improving SNR.

Methods have been proposed in the post-processing stage to remove structured noise arising from subtraction errors due to motion or other sources of difference between images while preserving as much signal as possible. Existing approaches include applying filters or removing image volumes deemed to be corrupted (Shirzadi et al., 2018; Tan et al., 2009). However, both strategies have their limitations. Filter thresholds are often chosen arbitrarily without reference to the imaging data. Removing entire volumes inevitably causes loss of signal, and risks being counterproductive in the presence of limited measurements.

The challenges of low SNR and the presence of structured noise are not unique to ASL. BOLD-based functional MRI (fMRI) shares the same issues. Using Independent Component Analysis (ICA) in post-processing BOLD fMRI data has been shown to reliably separate signal from artefacts or structured noise (Thomas et al., 2002), allowing a significant improvement over results obtained with more traditional post-processing (Stone et al., 2002; Kochiyama et al., 2005; McKeown et al., 2005; Zou et al., 2009). The utility of ICA to improve SNR has also been explored in diffusion-weighted imaging (Arfanakis et al., 2002), and dynamic susceptibility contrast-MRI (Calamante et al., 2004). It has shown promising preliminary results when applied to pre-clinical ASL data (Wells et al., 2010).

This study investigates the use of ICA-based denoising on clinical ASL data acquired in acute ischemic stroke patients. Its performance is compared to two other denoising strategies: aCompCor (Behzadi et al., 2007), a Principal Component-based method; and, Enhancement of Automated Blood Flow Estimates (ENABLE) (Shirzadi et al., 2018), an algorithm based on the removal of corrupted volumes.

## 2 Methods

### 2.1 Patients and MRI data acquisition

Consecutive patients presenting with a clinical stroke syndrome within 24 h of symptom onset (using the last seen well principle), regardless of age, were recruited into a prospective observational cohort study following informed consent or agreement from a representative according to protocols approved by UK National Research Ethics Service committees (ref: 12/SC/0292 and 13/SC/0362). Each subject was scanned at presentation, 24 h, a week, and a month later, whenever clinically possible. Exclusion criteria included the presence of a contraindication for MRI and a severely impaired conscious level (score >1 on question 1a of the National Institute for Health Stroke Scale).

#### 2.1.1 MRI data acquisition

All scans were acquired using a 3.0T Siemens Verio scanner (Siemens Healthcare, Erlangen, Germany). ASL data were acquired using the following protocol: pseudo-continuous ASL; single-shot EPI readout; TR/TE 5386/14 m s; 3.4x3.4 × 4.5 mm; 24 slices using a matrix size 64x64; alternating control and label pairs acquired after 1.8 s of labeling at 5 different post-labeling delays: 400, 800, 1200, 1600, 2000 m s, varied in a looped fashion and repeated 6 times (total volumes acquired 60); background suppression (WET presaturation and two global inversion pulses with timings calculated as per Okell et al. (2013)^6^); and, total acquisition time = 4 min 30 s. A calibration image with identical readout parameters, but with no background suppression or ASL labelling, was automatically collected within the same scan to allow quantification of CBF in absolute units. The vendor “pre-scan normalize” functionality used to remove the effects of receive coil non-uniformity.

In all patients and at all time points, a high-resolution T1-weighted structural image (magnetization prepared rapid acquisition gradient echo (MPRAGE); 1.8 × 1.8 × 1.0 mm; FoV = 228 mm; TR = 2040 m s; TE = 4.55 m s; and, a total acquisition time = 3 min 58 s) was also acquired.

### 2.2 Pre-processing

All image analysis was performed using tools from the Functional Magnetic Resonance Imaging of the Brain (FMRIB) Software Library (FSL 6.0, www.fmrib.ox.ac.uk/fsl). (Jenkinson et al., 2012)

All ASL datasets underwent motion correction via rigid-body registration using the MCFLIRT tool (Jenkinson et al., 2002), brain extraction (using BET (Smith, 2002)), and control-label subtraction (Chappell et al., 2009).

Tissue segmentation of the structural T1-weighted image using FMRIB’s Automated Segmentation Tool (FAST (Zhang et al., 2001)) defined gray matter partial volume estimates, which were registered into perfusion image space. Gray matter masks were generated using a partial volume estimate (PVE) threshold of ≥70% unless otherwise specified. Registration between perfusion and structural image was carried out using the BBR (Boundary-Based Registration) option of the FLIRT tool (Jenkinson et al., 2002; Jenkinson and Smith, 2001), which also allowed for simultaneous distortion correction using separately acquired fieldmaps. Registration between structural and standard-space (MNI152-2 mm standard brain) was carried out using the FNIRT tool (Jenkinson et al., 2012).

### 2.3 ICA-based denoising

Each 4D ASL dataset after control-label subtraction (ASL-sub) was processed using single-subject spatial-ICA decomposition with automatic dimensionality estimation using the Multivariate Exploratory Linear Optimised Decomposition of Independent Components (MELODIC) tool (Beckmann and Smith, 2004).

#### 2.3.1 Manual Independent Component classification and artefactual component regression

**Fig. 1:**
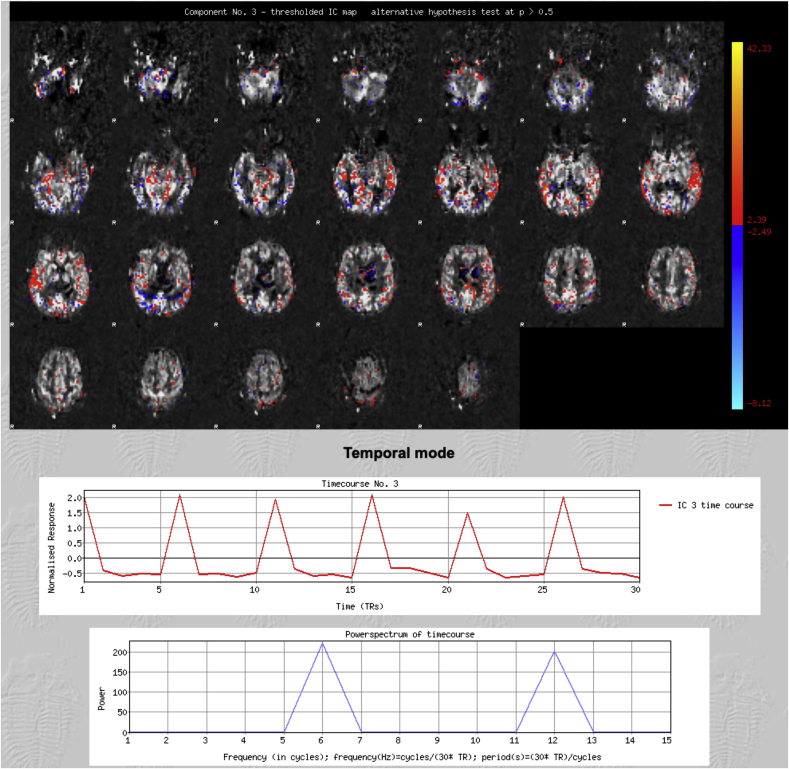
**Representative Signal component**. The spatial map (top) is consistent with the expected location of perfusion signal (i.e. gray matter); the time course (middle) is congruous with the post-labelling delays; most of the signal in the power spectrum (bottom) is at frequencies corresponding to the number of repetitions or its multiple. Patient age = 86 years.

Two independent raters manually classified the Independent Components (ICs) following a standardized procedure. Components were deemed as most likely representing signal (Fig. 1) when at least two of the following characteristics were present:

• spatial maps consistent with the expected location of perfusion signal (i.e. gray matter).
• a time course congruous with the variation in post-labeling delays across the acquisition.
• most of the signal in the power spectrum at frequencies (in cycles per scan) corresponding to the number of repetitions or its multiple.

**Fig. 2:**
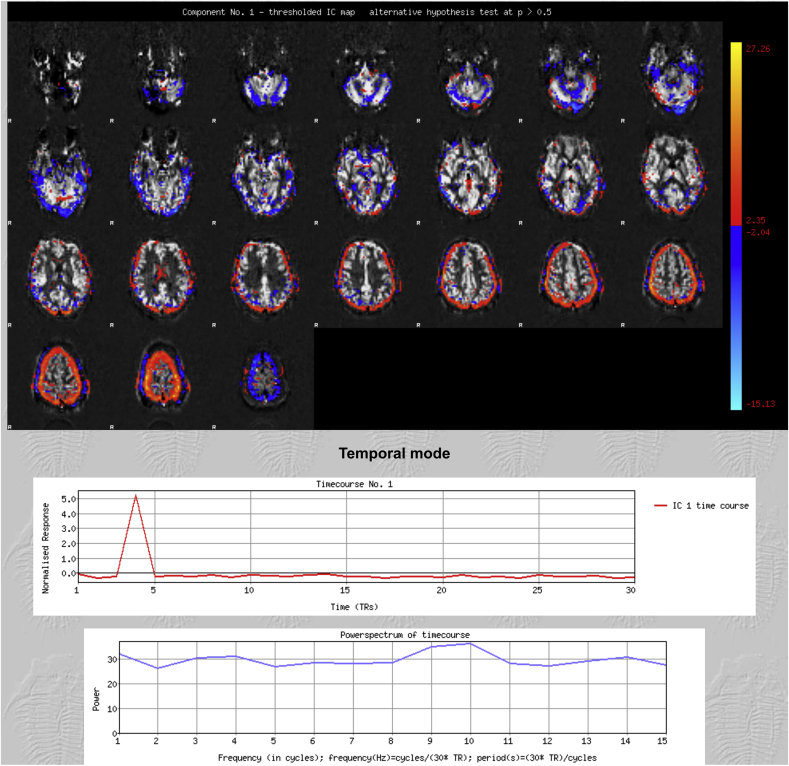
**Representative Noise component**. The spatial map (top) shows signal around the brain edges suggesting motion artefact. The time course is not consistent with the post-labelling delays. The signal in the power spectrum affects all frequencies equally.

If a component had none or only one of these characteristics, it was labeled as noise (Fig. 2), and regressed out of the data using a non-aggressive approach so that only the unique variance related to the artefacts was removed (Griffanti et al., 2014). Disagreements were resolved by reference to a third rater.

#### 2.3.2 Automated Independent Component classification and artefactual component regression

The automated classification and regression of components were obtained using a customized version of FMRIB’s ICA-based X-noiseifier (FIX) (Salimi-Khorshidi et al., 2014). FIX extracts multiple spatial and temporal features for each component, each describing a different aspect of the data. These are fed into a multi-level classifier. Once trained using manually classified training datasets, FIX can then automatically classify new datasets (Salimi-Khorshidi et al., 2014). A threshold is applied to FIX output to determine the binary classification of any given component. Changing the threshold shifts the balance between true positive ratio (TPR) and true negative ratio (TNR). For the purpose of this study, the optimal threshold was defined as the one that would grant the highest TNR while preserving a TPR above 90% (see Supplementary Table 1).

In order to optimize FIX for ASL data, it was custom-modified to identify as signal those ICs that had higher power in the frequencies that matched the specific ASL sequence PLD cycle frequency (6 cycles per scan). For more details on how FIX features were modified see Supplementary Table 2. In addition, FIX was automated to use the average motion parameters between the corresponding label and control images as motion parameters for each subtracted image. This was required as motion correction was performed before label-control subtraction, whilst ICA was run on the subtracted data.

The labels generated during manual classification were used to create the required training dataset. When denoising ASL data obtained from a specific subject, all the labels derived from the same subject were excluded from the training dataset (leave-one-subject-out approach). As above, components were automatically regressed out of the data using the non-aggressive approach.

### 2.4 Alternative denoising methods used for comparison

#### 2.4.1 aCompCor

aCompCor (Behzadi et al., 2007) is a Principal Component Analysis (PCA) based method developed to reduce noise in both BOLD and ASL based fMRI. Significant Principal Components are derived from noise regions-of-interest (ROI) composed primarily of white matter and cerebrospinal fluid. These components are then included as nuisance parameters within general linear models for BOLD and perfusion-based fMRI time-series data. aCompCor (available on https://nipype.readthedocs.io/en/latest/interfaces/generated/nipype.algorithms.confounds.html) was applied to the data used in this study after the pre-processing step described above in accordance with the user guide instructions.

#### 2.4.2 Enhancement of Automated Blood Flow Estimates (ENABLE)

ENABLE is a multiparametric automated algorithm that identifies and removes poor quality difference images in multiple post-labeling delay (PLD) ASL as a means to improve the SNR. The quality criteria that ENABLE simultaneously implements are: the temporal contrast to noise ratio (tCNR), defined as the temporal mean of the 4D ASL subtracted dataset (ASL-sub) in the gray matter divided by the standard deviation of ASL-sub in non-brain voxels); the proportion of ASL-sub voxels in the gray matter significantly greater than zero; the coefficient of variation (CoV, defined as the ASL-sub spatial standard deviation divided by its spatial mean in the gray matter; and, the temporal SNR (tSNR, defined as the spatial mean of ASL-sub divided by its standard deviation in the gray matter) (Shirzadi et al., 2018). ENABLE (available on https://asl-docs.readthedocs.io/en/latest/index.html) was applied to the data used in this study after the pre-processing step described above in accordance to the user guide instructions (Shirzadi et al., 2018).

### 2.5 Evaluation of the effects of denoising

**Effects on ASL-sub variance:** to test the effect of correction on ASL-sub, a voxel-wise variable, %ΔSTD_map_ (Carone et al., 2017; Khalili-Mahani et al., 2013) was calculated for every scan obtained. %ΔSTD_map_ was defined as:

%ΔSTD_map_ = (STD (ASL-sub_original_) − STD (ASL-sub_corrected_))/STD (ASL-sub_original_) × 100

where STD is the standard deviation of each voxel over the volumes of the 4D ASL subtracted dataset. The %ΔSTD maps were then registered to the MNI152-2 mm standard brain (using the non-linear transformation matrix obtained from registering the perfusion data to the MNI152-2 mm standard brain) and averaged to generate an intensity map. ΔSTD maps were also thresholded (25%), binarized, and averaged to generate a probability map that would highlight those areas the ASL-sub variance was more frequently reduced across subjects.

**Effects on the perfusion analysis:** gray matter CBF, ATT and their respective intrasession variances were estimated before and after denoising using a spatially regularized Bayesian inference method (BASIL) that produces an estimate of both CBF and its associated variance at every voxel (Chappell et al., 2009). Mean CBF and mean ATT and their respective intrasession variances were compared in the gray matter across the different denoising strategies using an ANOVA for repeated measurements with multiple comparisons. In addition, mean CBF and its intrasession variance were computed before and after ICA-based denoising using different gray matter PVE thresholds (50, 70 and 90) to explore the effects of denoising in regions with increasingly smaller partial volume effects.

BASIL-generated z-statistics for the CBF fit were used as a marker of goodness-of-fit to the model parameters (Chappell et al., 2009; MacIntosh et al., 2010). The number of gray matter voxels having a z value < 2 (approximately < 5% confidence in fitted value) was compared across the different denoising strategies using an ANOVA for repeated measurements with multiple comparisons.

**Effects on SNR estimates:** SNR was defined to allow the direct comparison of the results with previous work using ENABLE (Shirzadi et al., 2018). CBF-SNR and ATT-SNR were estimated for each dataset by dividing (voxel-wise) the gray matter CBF (or ATT) values by the estimated standard deviation. SNR estimates were compared across different denoising strategies using an ANOVA for repeated measurements with multiple comparisons.

**Effects on repeatability:** to understand the effect of denoising on repeatability of CBF measurements, each single session ASL dataset acquired was split into epochs comprising of one repetition for each post-labelling delay, before and after denoising. The gray matter CBF was estimated for each epoch. Repeatability was assessed using the coefficient of variation of these estimates (defined as the standard deviation divided by the mean; the lower the coefficient of variation, the higher the repeatability). The results were compared across different denoising strategies using an ANOVA for repeated measurements with multiple comparisons.

### 2.6 Evaluating the effects of varying the number of repetitions

Epochs were generated for each ASL dataset acquired, before and after manual ICA-based denoising. An increasing number of repetitions were removed in a stepwise manner to understand how varying the number of repetitions acquired may impact SNR (the first repetitions to be removed were the last acquired). CBF-SNR was then estimated for each epoch and compared using an ANOVA for repeated measurements with multiple comparisons.

### 2.7 Statistical analysis software

All statistical analysis was performed using Prism 8 (GraphPad, California, USA).

## 3 Results

### 3.1 Patients

72 scans were obtained from thirty consecutive patients with acute ischemic stroke (mean age 72 years (range 44–90); 18 females (43%)). Patient characteristics are summarised in Table 1.

**Table 1:** **Patient characteristics**. TACS = Total Anterior Circulation Stroke, PACS=Partial Anterior Circulation Stroke, LACS = Lacunar stroke. NIHSS = National Institutes of Health Stroke Scale at presentation. Pres = presenting.

Patient	Age	Sex	Bamford Classification	Hemisphere Affected	NIHSS	Pres. scan	24h scan	1-week scan	1-month scan
01	52	TACS	LACS	R	8	Y	N	Y	N
02	55	PACS	LACS	L	10	Y	N	Y	Y
03	71	LACS	PACS	R	7	Y	N	Y	N
04	90	TACS	TACS	R	10	Y	Y	Y	N
05	70	PACS	POCS	R	5	Y	Y	Y	Y
06	72	PACS	LACS	L	4	Y	Y	Y	Y
07	77	LACS	TACS	L	20	Y	N	N	N
08	82	PACS	PACS	R	8	Y	N	Y	Y
09	49	PACS	TACS	L	25	Y	N	N	Y
10	88	PACS	TACS	R	21	Y	Y	Y	N
11	55	LACS	TACS	L	21	Y	N	N	N
12	78	PACS	PACS	R	5	Y	Y	Y	Y
13	81	PACS	TACS	R	10	Y	N	Y	Y
14	60	TACS	PACS	L	3	Y	Y	Y	Y
15	81	PACS	LACS	R	10	Y	N	N	N
16	54	PACS	TACS	L	28	Y	N	N	N
17	84	PACS	PACS	L	2	Y	N	N	N
18	77	TACS	PACS	L	6	Y	N	N	N
19	56	LACS	LACS	L	4	Y	N	Y	Y
20	74	TACS	PACS	L	20	Y	Y	Y	N
21	80	TACS	PACS	R	5	Y	Y	Y	Y
22	77	POCS	PACS	L	8	Y	Y	N	N
23	90	TACS	LACS	R	4	Y	N	Y	N
24	84	LACS	PACS	L	3	Y	N	Y	N
25	72	LACS	PACS	R	13	Y	N	Y	N
26	78	POCS	TACS	R	15	Y	Y	Y	N
27	77	PACS	PACS	L	6	Y	Y	Y	N
28	82	LACS	PACS	L	11	Y	Y	Y	N
29	84	LACS	PACS	L	5	Y	N	Y	N
30	86	POCS	LACS	L	2	Y	N	Y	N

### 3.2 ICs classification and artefactual component regression

The mean number of single-subject ICs estimated by MELODIC was 19 (range 7–23).

The mean number of ICs manually classified as signal was 7 (range 3–14; inter-rater agreement ratio was 92%). The mean number of ICs classified as signal by FIX was 7 (range 2–16).

### 3.3 ENABLE

The mean number of volumes per patient scan classified as poor and subsequently removed from analysis was 1.7 (range 0–7).

### 3.4 Evaluation of the effects of denoising

**Fig. 3:**
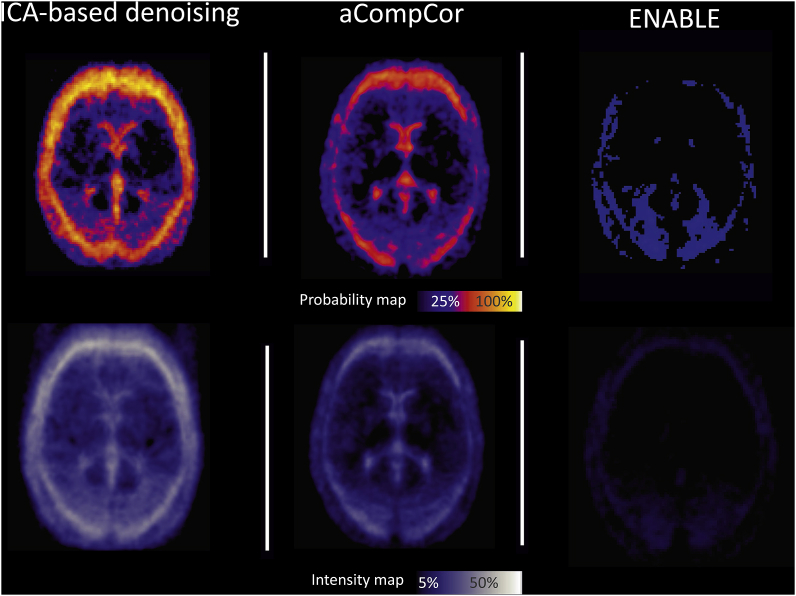
**Spatial pattern of change in ASL-sub variance after denoising**. Probability map (top), representing areas where the ASL-sub variance was affected more frequently in patients. Intensity map (bottom), representing average change in ASL-sub variance.

**Effect of correction on ASL-sub variance**: following ICA-based denoising, individual average maps of %ΔSTD showed more frequent and pronounced changes around the brain edges and in the periventricular areas with an average decrease of 30–35% in the ASL-sub variance (Fig. 3, right). When using aCompCor or ENABLE, the changes were similar although less frequent and less pronounced (Fig. 3, middle and left).

**Fig. 4:**
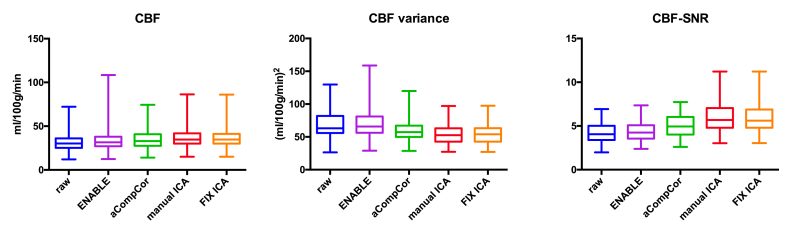
**Effects on CBF**. Gray matter mean CBF, CBF variance and CBF-SNR estimates. ICA-based denoising (manual and automated) led to significant increase in gray matter cerebral blood flow (CBF) estimates, significant decrease in CBF intra-session variance and to significant higher CBF-SNR. manual ICA = manual ICA-based denoising; FIX ICA = automated ICA-based denoising.

**Fig. 5:**
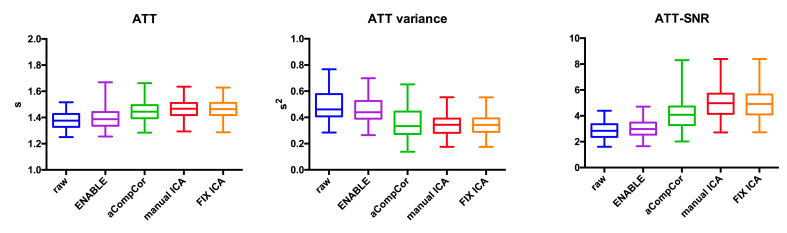
**Effects on ATT**. Gray matter ATT, ATT variance and ATT-SNR estimates. ICA-based denoising (manual and automated) led to significant increase in gray matter arterial transit time (ATT) estimates, decrease of ATT intra-session variance and to significant higher ATT-SNR. manual ICA = manual ICA-based denoising; FIX ICA = automated ICA-based denoising.

**Effects on the perfusion analysis**: the mean CBF and ATT values and their mean intra-session variances, before and after denoising are reported in Table 2 and Fig. 4, Fig. 5. Results from the multiple comparisons are listed in Table 3, Table 4.

**Table 2:** Group level mean CBF(ATT), CBF (ATT) intrasession variance, and CBF (ATT) SNR before and after denoising.

	CBF			ATT		
	Mean	Variance	SNR	Mean	Variance	SNR
Raw	31.8	68.8	4.2	1.38	0.49	2.9
ENABLE	33.9	70.4	4.3	1.39	0.46	3.1
aCompCor	34.9	59.8	5.0	1.45	0.36	4.2
MANUAL ICA	36.9	54.1	6.0	1.47	0.35	5.1
FIX ICA	36.7	54.7	5.9	1.46	0.35	5.0

**Table 3:** Results from the multiple comparison analysis (mean CBF, CBF intrasession variance, CBF-SNR). MANUAL ICA = manual ICA-based denoising; FIX ICA = automated ICA-based denoising.

Comparison	CBF			CBF variance			CBF-SNR		
	Mean Difference	95% CI	Summary	Mean Difference	95% CI	Summary	Mean Difference	95% CI	Summary
Raw vs. ENABLE	−2.1	−4.0 to −0.2	*	−1.6	−4.6 to 1.3	ns	−0.2	−0.3 to −0.1	***
Raw vs. aCompCor	−3.2	−3.9 to −2.4	****	9.0	6.2 to 11.8	****	−0.8	−0.9 to −0.7	****
raw vs. MANUAL ICA	−5.1	−6.1 to −4.2	****	14.7	10.4 to 18.9	****	−1.8	−2.1 to −1.5	****
raw vs. FIX ICA	−5.0	−5.9 to −4.1	****	14.1	9.8 to 18.4	****	−1.7	−2.0 to −1.5	****
ENABLE vs. aCompCor	−1.1	−3.1 to 1.0	ns	10.7	6.7 to 14.6	****	−0.7	−0.8 to −0.5	****
ENABLE vs. MANUAL ICA	−3.0	−4.6 to −1.5	****	16.3	11.1 to 21.5	****	−1.6	−1.9 to −1.4	****
ENABLE vs. FIX ICA	−2.9	−4.4 to −1.4	****	15.8	10.6 to 21.0	****	−1.6	−1.8 to −1.3	****
aCompCor vs. MANUAL ICA	−2.0	−3.3 to −0.7	***	5.7	2.2 to 9.1	***	−1.0	−1.2 to −0.7	****
aCompCor vs. FIX ICA	−1.8	−3.0 to −0.6	**	5.1	1.6 to 8.6	**	−0.9	−1.2 to −0.6	****
MANUAL ICA vs. FIX ICA	0.2	−0.1 to 0.4	ns	−0.5	−1.3 to 0.2	ns	0.08	−0.02 to 0.1	ns

**Table 4:** Results from the multiple comparison analysis (mean ATT, ATT intrasession variance, ATT-SNR). MANUAL ICA = manual ICA-based denoising; FIX ICA = automated ICA-based denoising.

Comparison	ATT			ATT variance			ATT-SNR		
	Mean Difference	95% CI	Summary	Mean Difference	95% CI	Summary	Mean Difference	95% CI	Summary
Raw vs. ENABLE	−0.01	−0.03 to 0.005	ns	0.03	0.01 to 0.04	***	−0.2	−0.30 to −0.05	**
Raw vs. aCompCor	−0.07	−0.09 to −0.04	****	0.13	0.12 to 0.14	****	−1.3	−1.6 to −1.0	****
raw vs. MANUAL ICA	−0.09	−0.10 to −0.07	****	0.14	0.12 to 0.16	****	−2.2	−2.5 to −1.9	****
raw vs. FIX ICA	−0.08	−0.10 to −0.07	****	0.14	0.12 to 0.16	****	−2.1	−2.4 to −1.8	****
ENABLE vs. aCompCor	−0.06	−0.09 to −0.03	****	0.10	0.08 to 0.12	****	−1.1	−1.5 to −0.8	****
ENABLE vs. MANUAL ICA	−0.08	−0.10 to −0.05	****	0.11	0.09 to 0.13	****	−2.0	−2.4 to −1.7	****
ENABLE vs. FIX ICA	−0.07	−0.09 to −0.05	****	0.11	0.09 to 0.13	****	−2.0	−2.3 to −1.6	****
aCompCor vs. MANUAL ICA	−0.02	−0.05 to 0.01	ns	0.009	−0.01 to 0.03	ns	−0.9	−1.3 to −0.5	****
aCompCor vs. FIX ICA	−0.01	−0.05 to 0.02	ns	0.007	−0.02 to 0.03	ns	−0.8	−1.2 to −0.4	****
MANUAL ICA vs. F IX ICA	0.004	−0.002 to 0.01	ns	−0.002	−0.004 to 0.0009	ns	0.08	−0.02 to 0.1	ns

ICA-based denoising led to the greatest changes in the perfusion analysis. Compared to both raw data and data denoised using the other methods, mean CBF was significantly higher (mean difference vs raw data: 5.0 ml/100 g/min, p < 0.001: vs aCompCor 1.8 ml/100 g/min, p < 0.01; vs ENABLE 2.8 ml/100 g/min, p < 0.001; Fig. 4, Table 3), whilst the mean CBF intra-session variance was lower (mean difference vs raw data: 21%, p < 0.001, vs aCompCor 9%, p < 0.01; vs ENABLE 18%, p < 0.001; Fig. 4, Table 3). There was no significant difference in either metric when the effects of manual and automated IC classification were compared. The use of aCompCor led to more modest changes in mean CBF (mean difference vs raw data: 3.2 ml/100 g/min; p < 0.001) and its intra-session variance (mean difference vs raw data: 17%, p < 0.001; Fig. 4, Table 3). The use of ENABLE resulted in an increase in mean CBF only (mean difference vs raw data: 1.5 ml/100 g/min, p < 0.05, Fig. 4, Table 3).

The use of ICA-based denoising and aCompCor led to similar changes in ATT (mean difference vs raw data: 0.08s, p < 0.001) and ATT intra-session variance (mean difference vs raw data: 28%, p < 0.001; Fig. 5, Table 4). There was no significant difference in either metric when the effects of manual and automated IC classification were compared. The use of ENABLE led to an increase in ATT intra-session variance only (mean relative difference: 5%, p < 0.001, Fig. 5, Table 4).

The changes in mean CBF and its intra-session variance following ICA-based denoising were significant across all gray matter PVE thresholds tested (see Supplementary Fig. 1).

All denoising strategies led to a significant decrease in the number of poorly fitted voxels (z value < 2) (Gardener and Jezzard, 2015). Compared to the raw data, this effect was greater following ICA-based denoising (14% decrease, p < 0.001, Supplementary Fig. 2). There was no significant difference between manual and automated ICA-based denoising. The effects of aCompCor and ENABLE were more modest but still significant (aCompCor: 10% decrease, p < 0.001; ENABLE: 8% decrease, p < 0.05).

**Effects on SNR estimates:** all denoising strategies led to a significant increase in both CBF- and ATT- SNR (p < 0.001 Fig. 4, Fig. 5; Table 2, Table 3). The highest increase in SNR compared to raw data was observed following ICA-based denoising (40% increase in CBF-SNR, p < 0.001, Fig. 4, Table 2; manual and automated). There was no significant difference between manual and automated ICA-based denoising (Fig. 4, Fig. 5, Table 2, Table 3). The effects of aCompCor were more modest (20% increase in CBF-SNR, p < 0.001, Fig. 4, Table 2) whilst the use of ENABLE led to the smallest increase (4% increase in CBF-SNR, p < 0.001, Fig. 4, Table 2, Table 3).

**Fig. 6:**
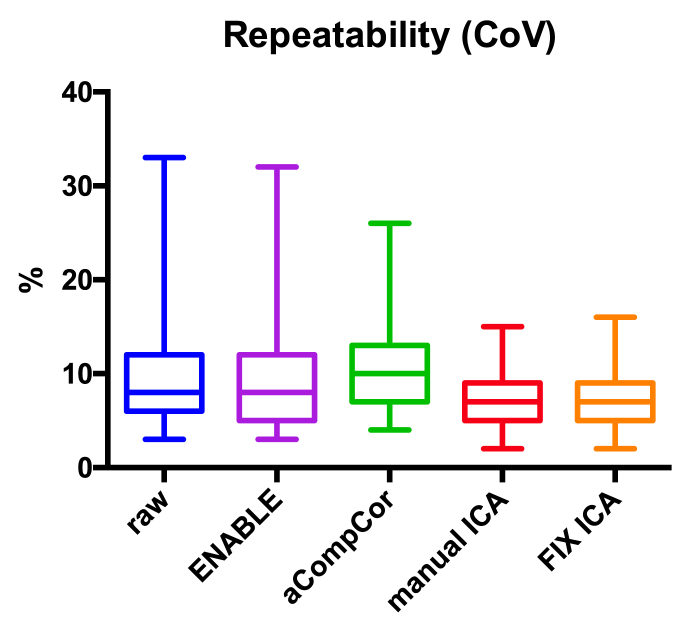
**Effects on Repeatability**. Data processed using ICA-based denoising (manual or automated) yielded significantly (p < 0.05) higher repeatability compared to raw data (the lower the coefficient of variability the higher the repeatability). manual ICA = manual ICA-based denoising; FIX ICA = automated ICA-based denoising.

**Effect of correction on repeatability:** repeatability was unaltered following the use of either aCompCor or ENABLE when compared to raw data. However, ICA-based denoising yielded significantly higher repeatability (*p* < 0.05, Fig. 6). There was no significant difference in repeatability between manual and automated ICA-based denoising (Fig. 6).

### 3.5 Evaluating the effects of varying the number of repetitions

**Fig. 7:**
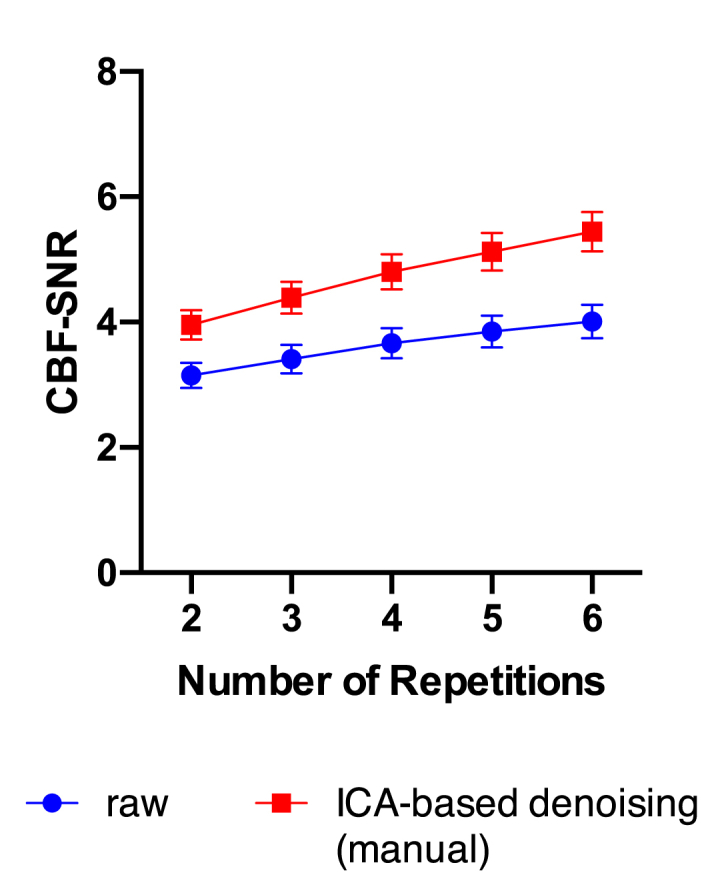
**Effects of varying number of repetitions**. Decreasing the number of repetitions in the led to a significant (p < 0.001) drop in CBF-SNR in both raw ASL-data and data denoised using ICA (manual). Apart from epochs comprising only two repetitions, the CBF-SNR calculated in denoised data was always greater than the CBF-SNR calculated from the complete raw data (p < 0.001 for epochs incorporating 3, 4, 5 and 6 repetitions).

Decreasing the number of repetitions in the raw ASL data led to a drop in CBF-SNR, ranging from 4% (one repetition removed, p < 0.001) to 21% (four repetitions removed, p < 0.001). A similar effect was observed when removing repetitions from the manual ICA denoised ASL data. However, the CBF-SNR calculated in denoised data incorporating only 3, 4, or 5 repetitions was greater than the CBF-SNR calculated from the raw data with all repetitions included (p < 0.001; Fig. 7).

## 4 Discussion

This study shows that the use of an ICA-based denoising approach results in lower CBF and ATT variance, increased SNR, and improved repeatability. These changes went beyond the effects of approaches using Principal Component Analysis (aCompCor) and removal of corrupt image volumes (ENABLE). Automating ICA denoising achieved near identical results to manual denoising across a broad range of metrics. Intriguingly, following ICA-based denoising, it was possible to obtain higher SNR levels compared to the full raw dataset using only 50% of the data acquired, setting up the possibility of reducing acquisition times in acute stroke patients.

An optimal approach to denoising ASL data should involve a simultaneous reduction in CBF and ATT variance alongside an improvement in repeatability with an associated increase in SNR (suggesting that the majority of signal has been preserved at the expense of noise removal). In this study, only the ICA-based approaches satisfied all these criteria. Further confidence that noise was removed was shown by the location of the changes in the ASL-sub variability matching expected sites of motion artefact (brain edges and periventricular areas). The increases seen in SNR were not only due to the reduction in variance, but also due to an increase in CBF and ATT mean value that was seen across all denoising strategies. This was, at least in part, a reflection of the reduction in the number of poorly fitted voxels; the Bayesian CBF and ATT priors used in BASIL are both less than the overall mean CBF and ATT, regardless of denoising strategy. As data fidelity improves, the prior has less influence over the overall CBF and ATT estimates (Chappell et al., 2009; Gardener and Jezzard, 2015).

Both component-based denoising approaches were superior to ENABLE presumably due to their ability to remove noise across multiple volumes without having to discard entire volumes containing both signal and noise. In settings where a small number of repetitions are acquired such as in this acute stroke study, ENABLE is disadvantaged as a denoising strategy as the concurrent removal of signal with noise has a greater marginal effect on the total signal available for analysis.

The comparative performance of the two component-based approaches was in line with previous studies comparing the two in BOLD fMRI where the denoising effects of aCompCor were more limited, especially in the presence of motion (Pruim et al., 2015; Parkes et al., 2018). This difference in effect appears to be related to inherent features of the approaches where different “rotation” criteria are used for determining the orientation of the basis vectors in their common solution space (James et al., 2009). PCA-based strategies are better in removing the contributions of random noise; whilst ICA-based approaches are better suited in dealing with structured noise such as motion, respiratory and cardiac noise (James et al., 2009; Biswal and Ulmer, 1999), major contributors of noise in acute stroke patients (Carone et al., 2017).

While manual IC classification has been widely used as the gold standard (De Martino et al., 2007; Rummel et al., 2013), it is time-consuming, operator dependent and requires expert knowledge to separate signal and noise characteristics (Carone et al., 2017; Salimi-Khorshidi et al., 2014). The customized version of FIX used here was able to achieve results comparable to manual ICA denoising, demonstrating that it is possible to automate this approach, overcoming these limitations. Moving to an automated approach may be challenging where the ASL data has been acquired with different scanners and/or different acquisition protocols. While the inherent structure of multi-PLD ASL data was exploited to help separate signal and noise components in this study, ICA denoising should also be beneficial in single-PLD ASL in the same it has been for BOLD fMRI. This requires further study. The customized version of FIX for ASL and our training datasets will be made available (URL).

## 5 Conclusion

ICA can be used to separate signal from noise in ASL data. The removal of artefactual components improves data quality without increasing acquisition duration. In fact, this study suggests that the acquisition time could be reduced by 50% without penalty to data quality, something that merits further investigation. Independent component classification and regression can be carried out either manually, following simple criteria, or automatically, thorough the use of FIX customized for ASL.
