# Supplementary material for: ICA-based denoising for ASL perfusion imaging
Source: Neuroimage. 2019 Oct 15;200:363–72. doi: 10.1016/j.neuroimage.2019.07.002 (PMC6711457; doi:10.1016/j.neuroimage.2019.07.002)
Supplement: Multimedia component 1 [file mmc1.docx]

**Supplementary Material**

| Threshold | 1 | 2 | 5 | 10 | 20 | 30 | 40 | 50 |
| --- | --- | --- | --- | --- | --- | --- | --- | --- |
| Median TPR | 100 | 100 | 100 | 100 | 100 | 100.0 | 83.3 | 80 |
| Median TNR | 23.5 | 33.3 | 42.9 | 54.5 | 71.4 | 80 | 86.7 | 89.5 |

Supplementary Table 1. **FIX independent component classification performance.**

TPR = true positive rate, i.e., the percentage of true signals correctly classified. TNR = true negative rate, i.e., the percentage of true artefacts correctly classified. As the FIX threshold is lowered, TPR is maximized at the expense of lower TNR.

| Feature Index | FIX  (original version) | FIX  (customized for ASL) |
| --- | --- | --- |
| 20–23 | The ratio of the sum of power above f_Hz_ to the sum of power below f_Hz_  for f = 0.1, 0.15, 0.2 and 0.25 | The ratio between N and D where  N = (sum of power between 0 and (PLDcf- f_cps_) ) + (sum of power between (PLDcf + f_cps_) and (2*PLDcf - f_cps_) ) + (sum of power between (2* PLDcf + f_cps_)  D = (sum of power between (PLDcf - f_cps_) and (PLDcf + f_cps_)) + (sum of power between (2*PLDcf - f_cps_) and (2*PLDcf + f_cps_))  for f = 1, 1.25, 1.5, 2 |
| 24–30 | Percent of total power that falls in 0:0.01, 0.01:0.025, 0.025:0.05, 0.05:0.1, 0.1:0.15, 0.15:0.2 and 0.2:0.25 Hz bins | Percent of total power that falls in 0:(PLDcf- 0.5_)_, (PLDcf- 0.5_)_: (PLDcf), (PLDcf):( PLDcf+ 0.5), (PLDcf+0.5): (2*PLDcf- 0.5), (2*PLDcf-0.5): (2*PLDcf), (2*PLDcf+ 0.5): (2*PLDcf+ 0.5) and (2*PLDcf+ 0.5): (3*PLDcf- 0.5) cps bins |

Supplementary Table 2. **Features modified in order optimize FIX for ASL.**

cps = cycles for scan, PLDcf = post labelling delay (PLD) cycle frequency (6 cps).

Supplementary Fig.1. **Effects of varying Partial Volume Estimates.** The changes in mean CBF and its variance following ICA-based denoising compared to raw data were significant using gray matter PVE thresholds of 0.50, 0.70, and 0.90. PVE=partial volume estimate.

Supplementary Fig.2. **Effects on the number of poorly fitted voxels.** All denoising strategies led to a significant decrease in the number of poorly fitted voxels (z value < 2). Compared to the raw data this effect was greater following ICA-based denoising (p<0.001). The effects of aCompCor and ENABLE were more modest but still significant (aCompCor p<0.001; ENABLE: p<0.05). manual ICA = manual ICA-based denoising; FIX ICA= automated ICA-based denoising.

Supplementary Fig3. **Additional Representative Signal components**. In all 3 cases the spatial map (top) is consistent with the expected location of perfusion signal (i.e. gray matter); the time course (middle) is congruous with the post-labelling delays; most of the signal in the power spectrum (bottom) is at frequencies corresponding to the number of repetitions or its multiple. Patient age, from left to the right=55,76 and 84 years.
